# Willingness of French General Practitioners to Prescribe mHealth Apps and Devices: Quantitative Study

**DOI:** 10.2196/28372

**Published:** 2022-02-11

**Authors:** Claire Della Vecchia, Tanguy Leroy, Charlotte Bauquier, Myriam Pannard, Aline Sarradon-Eck, David Darmon, Jean-Charles Dufour, Marie Preau

**Affiliations:** 1 Institut de Psychologie Université Lyon 2 Bron France; 2 Institut National de la Santé et de la Recherche Médicale Unité 1296 "Radiations: Defense, Health and Environment" Centre Léon-Bérard Lyon France; 3 Sciences Economiques & Sociales de la Santé & Traitement de l'Information Médicale Institut de Recherche pour le Développement Aix-Marseille Université Marseille France; 4 Institut Paoli-Calmettes CanBios Marseille France; 5 Département d'Enseignement et de Recherche en Médecine Générale Université Côte d'Azur Nice France; 6 Service Biostatistique et Technologies de l'Information et de la Communication Assistance Publique Hôpitaux de Marseille Hôpital de la Timone Marseille France

**Keywords:** mHealth, health applications, connected health and wellness devices, general practitioners, patients, prescription, quantitative study, mobile phone

## Abstract

**Background:**

The field of mobile health (mHealth) is constantly expanding. Integrating mHealth apps and devices in clinical practice is a major and complex challenge. General practitioners (GPs) are an essential link in a patient’s care pathway. As they are patients’ preferred health care intermediaries, GPs play an important role in supporting patients’ transition to mHealth.

**Objective:**

This study aims to identify the factors associated with the willingness of French GPs to prescribe mHealth apps and devices to their patients.

**Methods:**

This study was part of the ApiAppS project whose overall objective was to help remove barriers GPs face when prescribing mHealth apps and devices by developing a custom-built platform to aid them. The study included GPs recruited from the general practice department of several medical faculties in France (Lyon, Nice, and Rouen) and mailing lists of academic GPs, health care professional associations, and social and professional networks. Participants were asked to complete a web-based questionnaire that collected data on various sociodemographic variables, indicators of their involvement in continued education programs and the amount of time they dedicated to promoting healthy behaviors during patient consultations, and indicators characterizing their patient population. Data on their perceptions of mHealth apps and devices were also collected. Finally, the questionnaire included items to measure GPs’ acceptability of prescribing mHealth apps and devices for several health-related dimensions.

**Results:**

Of the 174 GPs, 129 (74.1%) declared their willingness to prescribe mHealth apps and devices to their patients. In multivariate analysis, involvement in continued education programs (odds ratio [OR] 6.17, 95% CI 1.52-28.72), a better patient base command of the French language (OR 1.45, 95% CI 1.13-1.88), GP-perceived benefits of mHealth apps and devices for both patients and their medical practice and GP-perceived drivers for mHealth apps and device implementation in their medical practice (OR 1.04, 95% CI 1.01-1.07), and validation of mHealth apps and devices through randomized clinical trials (OR 1.02, 95% CI 1.00-1.04) were all associated with GPs’ willingness to prescribe mHealth apps and devices. In contrast, older GPs (OR 0.95, 95% CI 0.91-0.98), female GPs (OR 0.26, 95% CI 0.09-0.69), and those who perceived risks for the patient or their medical practice (OR 0.96, 95% CI 0.94-0.99) were less inclined to prescribe mHealth apps and devices.

**Conclusions:**

mHealth apps and devices were generally seen by GPs as useful in general medicine and were, for the most part, favorable to prescribing them. Their full integration in general medicine will be conditioned by the need for conclusive certification, transparency (reliable and precise data concerning mHealth app and device methods of construction and clinical validation), software aids to assist GPs prescribe them, and dedicated training programs.

## Introduction

### mHealth Apps and Devices Worldwide

The World Health Organization defines mobile health (mHealth) as a “medical and public health practice supported by mobile devices, such as mobile phones, patient monitoring devices, personal digital assistants (PDAs), and other wireless devices” [1]. The area of mHealth continues to grow globally: in June 2021, there were over 350,000 health-related mobile apps worldwide, with more than 250 new apps being added to web-based stores every day [2]. In 2016, over 73 million connected health and wellness devices were sold worldwide. The report forecast a huge increase to 160 million devices sold in 2020 (the report on 2016-2020 data is not yet published) [3].

### The Prescribing of mHealth Apps and Devices

Although the feasibility of prescribing mHealth apps and devices in general practice, in Australia [4] and Spain [5] notably, has been demonstrated, their integration in clinical practice worldwide presents a complex challenge. In France, the various physician organizations agree on the role of the general practitioner (GP) in health care, especially to provide primary care based on a comprehensive approach that includes providing advice and support focusing on education, risk prevention, and health promotion. GPs also play a role in monitoring and coordinating patient care (guaranteeing communication between themselves and other professionals involved in their patients’ care) [6]. GPs are essential links in the patient’s care pathway [7,8]. According to a 2018 French general population study, 83% of French people consulted a GP at least once a year [9] and 90% of health problems are managed in primary care (especially in general practice) [10]. As GPs are patients’ preferred health care intermediaries, they play a key role in patient support and patients’ relationship with mHealth, especially by providing guidance and advice. To promote the full implementation and acceptance of mHealth in general medicine, it is necessary to consider upstream both human (attitudes, expectations toward mHealth, and the characteristics of GPs and their patient base) and technical implications (mHealth apps and device functionalities, ease of use, ease of data transfer, operability, compatibility with electronic medical records and computer software used by GPs, etc) [11]. In France, to facilitate the integration of mHealth, the ApiAppS project aims to propose a type of software (considering the perceptions GPs have toward mHealth) in primary care to help GPs prescribe mHealth apps and devices adapted to the patient’s condition and provide reliable information regarding mHealth apps and devices [11].

### The Prescribing of mHealth Apps and Devices in the French Context

The national organization of French physicians published a report in 2015 that defined recommendations for good practices in mHealth app and device use [12]. The report indicated that mHealth apps and devices must support care to strengthen prevention behaviors, improve care monitoring and coordination, strengthen the patient–physician relationship, enable better access to care, and promote the empowerment of patients [12]. These same elements were highlighted in a French study investigating the drivers for the use of mHealth apps and devices in general medicine [13]. In this study, beyond the simple (informal) recommendation, it seemed interesting to investigate the potential prescription of mHealth apps and devices. In France, physicians are responsible for writing the prescription and making sure to give all the necessary information to the patient or their entourage to ensure proper compliance and the correct use of the prescribed elements [14]. The prescription is thus much more binding for physicians than a simple recommendation, constituting a material symbol of the patient–physician relationship [15] and the document required for reimbursement by the French social security health care insurance. In France, some connected health devices are reimbursed by social security health care insurance, especially devices for the management of diabetes, coagulation disorders, sleep apnea, and asthma. However, to our knowledge, only 2 mHealth apps—one for monitoring diabetes and the other for lung cancer—can currently be prescribed by physicians and then reimbursed by French social security health care insurance.

### Risks and Obstacles Linked to the Use of mHealth Apps and Devices

Although there are many potential advantages of mHealth apps and devices, their methods of construction, validation, and uses must be regulated. The international literature highlights the various primary types of risks and obstacles linked to the use of mHealth apps and devices in the following areas: data processing (data security and the use of personal data), reliability (lack of clinical validation, evaluation, precision of measurements, and reliable sources listing mHealth apps and devices) [12,13,16-19], the impact on patient care, and quality of the patient–physician relationship [13,20-22]. Physicians have also reported potential obstacles directly linked to their practice, in terms of the additional time spent during consultations processing digital information and providing patients support in the use of mHealth apps and devices [13,16,21,22], as well as the risk that the current divide between digitally literate and illiterate patients will become even greater, something that could increase inequalities in quality and access to care [13,16].

### GPs’ Perceptions of mHealth Apps and Devices and Willingness to Prescribe

To date, few studies have investigated the perceptions of GPs about mHealth apps and devices and how these beliefs are associated with their willingness to prescribe mHealth apps and devices to their patients. This way, a qualitative study was conducted with French GPs with the aim of investigating their attitudes toward the prescription of mHealth apps and devices. They identified 3 groups of attitudes. The first group corresponds to GPs very willing to prescribe mHealth apps and devices, with positive perceptions of (1) the benefits for patients and for clinical practice and (2) ease of use; the second group represents GPs worried about the protection of patient data and the reliability of mHealth app and device content; and the third group corresponds to GPs concerned about the implications of mHealth apps and devices for their clinical practice (additional working time, modification of the patient–physician relationship, and the importance of mHealth app and device certification by independent entities) [23]. Consistently, an Australian study showed that the perceived barriers of GPs to prescribing mHealth apps and devices were generational digital divide, a lack of knowledge and reliable resources listing prescriptible mHealth apps and devices, additional working time it may represent for GPs, and concerns about data security [4]. To our knowledge, most studies have focused on factors associated with the intentions of physicians and other health care professionals to use mHealth apps and devices to support their own clinical practice (drug database, medical calculators, making appointments, etc) or studies based on informal recommendation (mostly oral) of mHealth interventions to their patients. Fewer studies focused on mHealth apps and devices intended for prescription or its equivalent in some contexts (formal and written recommendation), more binding for GPs and their patients. One study conducted among Turkish physicians showed that a manifest interest in mHealth apps and devices, very little fear about using them, perceiving mHealth apps and devices as useful for medical practice, and ease of access for physicians were associated with an increased willingness to use mHealth apps in medical practice [24]. These results were corroborated by 2 other studies conducted with Chinese health care professionals [25,26]. Unfortunately, none of these 3 studies provided much information on the determinants of prescribing mHealth apps and devices to patients in medical practice, as they focused on mHealth app and device acceptance by health care professionals (especially mHealth apps and devices for their own practice) and not on mHealth app and device prescription purposes. However, we found a study that investigated factors associated with the willingness of Malaysian GPs to recommend mHealth apps to their patients. They showed that in multivariate analysis, performance expectancy of the mHealth apps (improving patient health, improving chronic disease management, and encouraging patients to gain health knowledge) was associated with the willingness of GPs to recommend mHealth apps [27]. However, this study focused only on the mHealth app recommendation, which may have different implications for mHealth app and device prescription. A recent descriptive study was conducted to better understand German GPs’ perceptions of mHealth apps. Of the 2138 GPs, although 60% recognized that mHealth apps could strengthen the involvement of people in the management of their health, only 36% reported global positive opinions of the health apps and only 18% frequently recommend mHealth apps to their patients, and the main criteria reported to recommend these apps were ease of use, guarantees for data privacy, and clinical validation [28]. However, this study focused only on mHealth app recommendation (not prescription), remained descriptive, thus results have to be corroborated by analytic studies.

### Objectives

On the basis of data from the literature, it seems essential to quantitatively describe GPs’ perceptions of mHealth apps and devices and investigate the factors associated with the willingness of French GPs to prescribe mHealth apps and devices to their patients, constituting the objective of this study. This way, we hypothesize in a psychosocial perspective that characteristics related to GPs themselves, their practice, their patient base, and their perceptions of mHealth apps and devices may influence their willingness to prescribe mHealth apps and devices.

## Methods

### Study Design and Study Population

This study adheres to and has been reported following the Checklist for Reporting Results of Internet E-Surveys guidelines (Multimedia Appendix 1) [29].

This quantitative study was part of a larger project called ApiAppS (funded by the National Research Agency of France under grant ANR-17-CE19-0027 [11]), whose overall objective is to help remove barriers GPs face when prescribing mHealth apps and devices by developing a custom-built platform to aid them. This study aimed to confirm the results of a previous exploratory qualitative study [23], which investigated the attitudes of French GPs about prescribing patient-based mHealth apps and devices by analyzing their perceptions and expectations of mHealth apps and devices through semistructured interviews and focus groups [23].

For this quantitative study, we constructed a web-based, self-administered questionnaire on the basis of the results of the qualitative study concerning attitudes of GPs toward the prescription of mHealth apps and devices [23] and elements from the literature concerning mHealth apps and devices in current clinical practice. The various indicators measured by the questionnaire are described in detail in subsequent sections. The questionnaire was pretested with 8 GPs regarding the understanding of the different items in the questionnaire, and the researchers tested the technical functionalities of the questionnaire (any technical problems with posting the questionnaire on the web-based platform: no glitches in the layout of the questions and answers, the sequencing of the questions, and the recording of the questionnaire). These 2 test phases made it possible to correct, where necessary, the layout of the questionnaire (spelling, fonts, order of questions, etc) and the obligatory or nonobligatory nature of each question. From June 2019 to December 2019, the questionnaire was then distributed to GPs recruited through several academic departments of general practice of several medical faculties in France (Lyon, Nice, and Rouen) and also from mailing lists of the academic GPs, health care professional associations, and social and professional networks. Participation in the study was voluntary and required a survey link, which headed toward the questionnaire (on the LimeSurvey platform). Information was provided about the time needed to fill in the questionnaire (about 15 minutes, 22 items), reminders about the rights of research participants under French law (anonymity, confidentiality, processing of data for research purposes, right of access, and data rectification), and email addresses of the researchers in charge of the study were provided. Once participants had validated their answers to the questionnaire, they could no longer review and change their answers. Only fully completed questionnaires were analyzed.

Ethical approval was obtained from the French Institute of Medical and Health Research Ethics Committee (IORG0003254 and FWA00005831) and the institutional review board (IRB00003888; opinion number 18-499).

### Questionnaire

The questionnaire, available in Multimedia Appendix 2, for this study included sociodemographic variables relating to GPs, indicators of their participation in continuing education programs, and the amount of time they dedicated to promoting healthy behaviors to their patients during consultations. It also included variables that allowed us to characterize the patient population. Finally, to meet the objective of this study, the questionnaire included variables aimed at gathering a greater understanding of GPs perceptions of mHealth apps and devices.

The objective of this study is to predict the willingness of GPs to prescribe mHealth apps and devices for twelve health-related dimensions: physical activity, dental health, nutrition, vaccination, sexual and reproductive health, well-being and mental health, addictions, asthma and allergies, dermatology, diabetes, first aid, and support for caregivers. For the analysis, to oppose 2 profiles of GPs, willingness to prescribe mHealth apps and devices was dichotomized into willingness and unwillingness to prescribe mHealth apps and devices for at least one health-related dimension.

Potential predictive variables were sociodemographic factors (age and gender) and factors related to GPs practices (indicators of GP involvement in continuous medical education, including subscription to professional journals, participation in peer groups, training, and presence in physician-based social networks), and the amount of time they dedicated during consultations in promoting healthy behaviors. We also considered psychosocial variables to help characterize the patient population as follows: the mean age of the practice population in the previous month; the place of residence; and the perceptions of GPs of the overall socioeconomic status, command of the French language, and self-management skills in terms of health of the patient base. On the basis of the previous qualitative study regarding attitudes GPs have toward mHealth app and device prescriptions [23], their perceptions of mHealth apps and devices were investigated as other potential predictors in our analysis:

- Facilitators regarding mHealth apps and devices implementation in general medicine, that is, both the potential perceived benefits of mHealth apps and devices and the levers to their implementation. This indicator included providing better access to care for patients, patient empowerment, better communication, quality of life, and work management for caregivers; obtaining additional information from patients (Patient-Reported Outcome Measures [30]); and facilitating links between the various professionals involved in patient care, an alternative to prescribing drugs, the strengthening of the patient–physician relationship, the perception of the importance of the role of the physician in the transition to mHealth, and the possibility of having a software aid that would automatically suggest mHealth apps and devices adapted to the needs of the patient.
- Obstacles to the implementation of mHealth apps and devices in general practice, that is, the risks and barriers associated with the use of mHealth apps and devices. This indicator included the dangers linked to misuse of mHealth apps and devices by patients, risks associated with self-medication, dehumanization of the patient–physician relationship, increase in patient anxiety because of the wealth of information available, use of personal data of patients, possibility of monitoring activities of GPs by health authorities, and devotion of additional time to mHealth apps and devices during consultations.
- Indicators relating to GPs perception of the importance of the development, clinical validation, and certification of mHealth apps and devices by GP-perceived trusted actors in health (eg, independent experts, patients’ associations, academic researchers, physicians, health-related organizations, and stakeholders). Furthermore, GPs’ perceptions of the importance of the involvement of health-related organizations and stakeholders in promoting the use of mHealth apps and devices in general medicine.

Scores on these indicators ranging from 0 to 100, with 100 representing the greatest perceived benefit or driver, risk or barrier, involvement, or utility, as applicable.

### Data Analyses

Several principal component analyses were performed to identify the underlying structure of data and highlight indicators (by grouping items belonging to the same 1D construct to generate a score). Specifically, for each component, the eigenvalues were extracted to capture the percentage of inertia explained by the component. Those greater than 1 (Kaiser criterion) were retained [31]. The choice of components was compared with the graph of the eigenvalues [32]. Finally, the results of these 2 methods were compared using parallel analysis to retain only those components that made the most sense at the theoretical level [33]. Internal consistency was assessed by calculating the Cronbach α coefficient [34]. All created indicators (patients’ skills in self-management of their health, facilitators and obstacles to mHealth implementation, importance of involvement of trusted actors in health in the construction of mHealth apps and devices, usefulness of mHealth apps and devices certification, and importance of the involvement of health-related organizations and stakeholders in promoting the use of mHealth in general medicine) were obtained by adding up the scores for each item in the indicator and converting them into a score from 0 to 100, with 100 representing the greatest perceived benefit, risk or barrier, involvement, or utility, as applicable.

Descriptive analyses of the variables in the sample were then performed, followed by a multivariate binomial logistic regression to investigate the willingness of GPs to prescribe mHealth apps and devices. To select the variables to be included in the multivariate model, we performed univariate logistic regressions, which made it possible to obtain the crude odds ratios (ORs) and their 95% CIs and the *P* value. Variables associated with a 20% *P* value threshold (*P*<.20) in the univariate analyses were retained in the final multivariate model [35]. Once the latter was established, we verified that there was no problem with multicollinearity, defined as a variance inflation factor greater than 2.5 [36]. To obtain the most efficient and parsimonious model reflecting our data, a stepwise selection combining forward and backward selection procedures was performed. The model with the lowest Akaike Information Criterion was retained. We compared this model with the starting model (variables significant at the 20% *P* value threshold in univariate analyses) using analysis of variance. The multiple logistic regression coefficients were presented as adjusted ORs with their 95% CIs. We tested interactions between GPs’ gender and facilitators and obstacles in the model as it was shown in the general population that gender was a moderator between attitudes toward mHealth apps and the intention to use them [37]. To estimate the goodness of fit of the model, the McFadden pseudo-*R*^2^ value was calculated. In addition, to assess the discrimination of the model, the area under the receiver operator characteristic curve was also determined. The level of significance for the multivariate model was set at the 5% *P* value threshold. Analyses were performed using RStudio (version 1.2.5033; RStudio Inc) [38].

## Results

### Sociodemographic Characteristics of the GPs and Their Patients

Among the 226 GPs who answered the first question of the survey, 174 (76.9%) fully completed the questionnaire. The study sample comprised thus 174 GPs. Almost two-thirds (112/174, 64.4%) were men, and the mean age was 45.1 (SD 13.0) years. Nearly half of the GPs (80/174, 45.9%) reported spending 40% or more of their consultation time promoting healthy behaviors. With regard to their patient base, 37.4% (65/174) of GPs declared having patients mainly aged between 45 and 69 years in the previous month. One-third (58/174, 32.8%) reported that their patient base was made up of people of different ages. Approximately, as many patients came from an urban setting as from a rural setting. Participating GPs estimated that, overall, their patient base had a middle socioeconomic status and quite a good command of the French language. However, in terms of self-management of their health, GPs perceived the skills of patients to be quite modest, with an average score of 49 (SD 15.3; Table 1).

**Table 1:** Sociodemographic characteristics of general practitioners (GPs) and their patients; characterization of GP practice and their perception of the self-management skills of patients in terms of health (N=174).

Variables		Values
Age of GP (years), mean (SD)		45.1 (13.0)
**Gender of GP, n (%)**		
	Male	112 (64.4)
	Female	62 (35.6)
**Time spent promoting good health behaviors during consultations (consultation %), n (%)**		
	0-20	45 (25.9)
	20-30	49 (28.2)
	30-50	49 (28.2)
	50-100	31 (17.8)
**Participation in a continued education program during the previous year, n (%)**		
	No	14 (8)
	Yes	160 (91.9)
**Participation in a peer group during the previous year, n (%)**		
	No	93 (53.4)
	Yes	81 (46.6)
**Subscription to a professional magazine, n (%)**		
	No	34 (19.5)
	Yes	140 (80.5)
**Part of a social network for physicians, n (%)**		
	No	105 (60.3)
	Yes	69 (39.7)
**Age of patients in the previous month (years), n (%)**		
	0-44	31 (17.8)
	45-69	65 (37.4)
	70 and older	20 (11.5)
	Other (not characterizable)	58 (33.3)
Patient base place of residence (GPs perceived): urban setting (0)-rural setting (6), mean (SD)		3.3 (2.1)
Patient base socioeconomic status (GPs perceived): low (0)-high (6), mean (SD)		3.4 (1.5)
Patient base command of the French language (GPs perceived): poor (0)-excellent (6), mean (SD)		4.6 (1.6)
Patient skills in self-management of their health^a^ (GPs perceived; prevention behaviors, autonomous health management, and assessment of the reliability of information): low (0)-high (100), mean (SD)		49 (15.3)

^a^Internal consistency (Cronbach α)=.80.

### GPs and mHealth Apps and Devices

Participating GPs were more likely to have mHealth apps (mainly for mixed personal and professional use) than connected health and wellness devices (132/174, 75.9%, vs 84/174, 48.3%, respectively; Table 2).

**Table 2:** Participating general practitioners (GPs) and mobile health (mHealth) apps and devices (possession and perceptions; N=174).

Variables			Values		Internal consistency (Cronbach α)
**Had a connected health or wellness device, n (%)**					—^a^
	No	90 (51.7)			
	Yes	84 (48.3)			
**Had an mHealth app, n (%)**					—
	No	42 (24.1)			
	Yes	132 (75.9)			
Facilitators: perceptions of GPs of the benefits of mHealth apps and devices for patients, caregivers, their own clinical practice, and GP-perceived drivers for mHealth apps and devices implementation in their medical practice^b^, mean (SD)			57.2 (16.6)		.91
Obstacles: perceptions of GPs of risks for the patient and barriers for the GPs practice^b^, mean (SD)			54.1 (15.6)		.71
Perceptions of GPs of the importance of the involvement of trusted actors in health in the construction of mHealth apps and devices^b^, mean (SD)			75.5 (19.8)		.76
Perceptions of GPs of the usefulness of mHealth apps and devices certification^b^, mean (SD)			64.2 (15.3)		.71
Perceptions of GPs of the importance of the involvement of health-related organizations and stakeholders in promoting the use of mHealth apps and devices in general medicine^b^, mean (SD)			64.6 (22.7)		.78
Perceptions of GPs of the utility of validation of mHealth apps and devices using randomized studies (evidence-based medicine)^b^, mean (SD)			81.1 (21.4)		—

^a^Cronbach α could not be estimated because of qualitative variables or a single quantitative item.

^b^Score ranging from 0 to 100, with 100 representing the greatest perceived benefit or driver, risk or barrier, involvement, or utility, as applicable.

### Perceptions of GPs of the Benefits and Drivers of mHealth Apps and Device Prescriptions and the Associated Risks and Barriers

GPs perceived as many benefits and potential drivers to mHealth apps and devices use by their patients (mean 57.2, SD 16.6) as they did risks and barriers (average score 54.1, SD 15.6, out of a possible score of 100; Table 2). More specifically, regarding benefits and potential drivers to mHealth devices implementation, the higher perception was that their patients would use mHealth apps and devices more if they recommended it (mean 5.3, SD 1.3, out of a possible score of 7), followed by the perception that mHealth apps and devices could strengthen the involvement of patients in the management of their health (average score 5.1, SD 1.1) and by an alternative to drug prescription (average score 4.9, SD 1.4). A wish for access to a software aid that could help them prescribe mHealth apps and devices (ie, software that would automatically suggest mHealth apps and devices adapted to the patient’s needs) was expressed by GPs as facilitators (average score 4.9, SD 1.8). It is relevant to note that GPs reported a low level of knowledge regarding mHealth apps and devices (average score 3.0, SD 1.6; Figure S1 in Multimedia Appendix 3).

With regard to the perceived risks and barriers, their main concern (average score 5.5, SD 1.2, out of a possible score of 7) was that GPs must provide support in the use of mHealth apps and devices, meaning additional working time during and outside of consultations. This concern was followed by their fear that patient data would be used for commercial reasons (average score 5.4, SD 1.7). It should be noted that GPs shared low levels of concern regarding the risk of dehumanization of the patient–physician relationship (average score 3.3, SD 1.6; Figure S2 in Multimedia Appendix 3).

### GPs Perceptions of the Construction, Validation, and Certification of mHealth Apps and Devices

GPs reported a high importance of the implication of trusted actors in health in the construction of mHealth apps and devices (average score 75.5, SD 19.8, out of a possible score of 100). Precisely, GPs who participated in the study considered the involvement of physicians (average score 5.9, SD 1.3; out of a possible score of 7) and patients (average score 5.7, SD 1.4; out of a possible score of 7) in the construction and development of mHealth apps and devices content to be necessary. The average score was 5.0 (SD 1.5) when asked about the involvement of researchers, and the average score was 5.0 (SD 1; Figure S3 in Multimedia Appendix 3). GPs also underlined their strong belief that mHealth apps and devices should be clinically validated through randomized studies (average score 81.1, SD 21.4; out of 100) and obtain certification from trusted health actors (average score 64.2, SD 15.3; out of 100). More specifically, certification by independent experts, a college of physicians, or an ethics committee was necessary and even essential (average score 5.8 out of 7, SD 1.4 for the 3 items). University certification (average score 5.0, SD 1.5) or patients’ association certification (average score 4.8, SD 1.7) were also considered necessary. Conversely, GPs reported relatively unnecessary certification by private health companies (average score 1.9, SD 1.2; Figure S4 in Multimedia Appendix 3). The issues surrounding clinical validation and certification raise the question of financial implications. Implications of health-related organizations and stakeholders in promoting the use of mHealth apps and devices in general medicine were reported as an important issue (average score 64.6, SD 22.7; out of 100). Precisely, GPs considered it necessary to cover the costs of mHealth apps and devices by patient health care insurance or complementary health insurance firms (mean 5.0, SD 1.7; for both) and reported that it was necessary for health authorities to provide a financial incentive to GPs to prescribe mHealth apps and devices (average score 4.3, SD 2.0). The involvement of health authorities (French National Authority for Health) was clearly reported by GPs as a driver for the implementation of mHealth apps and devices in general practice, with an average score of 5.1 (SD 1.6) out of 7 (Figure S5 in Multimedia Appendix 3; Table 2).

### Willingness of GPs to Prescribe mHealth Apps and Devices

Of the 129 GPs, 97 (75.2%) were willing to prescribe mHealth apps and devices, that is, they were willing to prescribe mHealth apps and devices for at least one of the 12 health dimensions included in this study. More specifically, 60.5% (78/129) declared their willingness to prescribe mHealth apps and devices for physical activity, asthma and allergies, and vaccination; 79.8% (103/129) for diabetes; and 71.3% (92/129) for addictions (Multimedia Appendix 4).

### Univariate Binomial Logistic Regressions

Table 3 shows that at the 5% *P* value threshold, the following factors were associated with the willingness of GPs to prescribe mHealth apps and devices: having participated in a training program during the previous year (OR 6.20, 95% CI 2.01-21.28); having participated in a peer group during the previous year (OR 2.10, 95% CI 1.04-4.35); and GPs’ perception that, overall, their patient base had a good command of the French language (OR 1.24 95% CI 1.01-1.51), facilitators of mHealth apps and devices implementation (perception of benefits and drivers; OR 1.04 95% CI 1.02-1.06). Conversely, GPs’ age (OR 0.97, 95% CI 0.95-0.99) and their perception of risks for the patient and obstacles to their own practice (OR 0.97, 95% CI 0.95-0.99) were associated with unwillingness to prescribe mHealth apps and devices.

At the 20% *P* value threshold, subscribing to a professional journal (OR 1.77, 95% CI 0.77-3.91), owning a connected health or wellness device (OR 1.78, 95% CI 0.90-3.62), perceiving that the involvement of field-based actors in developing mHealth apps and devices is important (OR 1.02, 95% CI 1.00-1.03), and perceiving that validation of mHealth apps and devices by randomized studies is necessary (OR 1.01, 95% CI 1.00-1.03) were associated with greater willingness to prescribe mHealth apps and devices. In contrast, being a female GP (OR 0.60, 95% CI 0.30-1.21) was associated with the unwillingness to prescribe mHealth apps and devices (Table 3).

**Table 3:** Characteristics of general practitioners (GPs) and their patients, attitudes GPs have toward mobile health (mHealth) apps and devices, and their association with the willingness of GPs to prescribe mHealth apps and devices in univariate analyses (N=174).

Variables		Unwilling to prescribe mHealth apps and devices (n=45)	Willing to prescribe mHealth apps and devices (n=129)	Crude OR^a^ (95% CI)	*P* value	
Age of GPs (years), mean (SD)		48.7 (13.8)	43.8 (12.6)	0.97 (0.95-0.99)	.03	
**Gender of GPs, n (%)**						.15
	Male	25 (55.6)	87 (67.4)	Ref^b^		
	Female	20 (44.4)	42 (32.6)	0.60 (0.30-1.21)		
**Time spent promoting healthy behaviors during consultations (% of consultation), n (%)**						.24
	0-20	9 (20)	36 (27.9)	Ref		
	20-30	16 (35.6)	33 (25.6)	0.52 (0.19-1.30)		
	30-50	15 (33.3)	34 (26.4)	0.57 (0.21-1.45)		
	50-100	5 (11.1)	26 (20.1)	1.30 (0.40-4.65)		
**Participation in a continued education program during the previous year, n (%)**						.002
	No	9 (20)	5 (3.9)	Ref		
	Yes	36 (80)	124 (96.1)	6.20 (2.01-21.28)		
**Participation in peer group during the previous year, n (%)**						.04
	No	30 (66.7)	63 (48.8)	Ref		
	Yes	15 (33.3)	66 (51.2)	2.10 (1.04-4.35)		
**Subscription to a professional magazine. n (%)**						.17
	No	12 (26.7)	22 (17.1)	Ref		
	Yes	33 (73.3)	107 (82.9)	1.77 (0.77-3.91)		
**Part of a social network for physicians, n (%)**						.77
	No	28 (62.2)	77 (59.7)	Ref		
	Yes	17 (37.8)	52 (40.3)	1.11 (0.56-2.27)		
**Age of patient base in the previous month (years), n (%)**						.71
	0-45	7 (15.6)	24 (18.6)	1.12 (0.42-3.24)		
	45-70	16 (35.5)	49 (38)	Ref		
	≥70	4 (8.9)	16 (12.4)	1.31 (0.41-5.06)		
	Other (not characterizable)	18 (40)	40 (31)	0.73 (0.33-1.60)		
Patient base place of residence (GP perceived): urban setting (0)-rural setting (6), mean (SD)		3.6 (2.3)	3.2 (2.1)	0.92 (0.78-1.08)	.32	
Patient base socioeconomic status (GP perceived): low (0)-high (6), mean (SD)		3.2 (1.6)	3.5 (1.4)	1.14 (0.91-1.44)	.25	
Patient base command of the French language (GP perceived): poor (0)-excellent (6), mean (SD)		4.2 (1.8)	4.8 (1.5)	1.24 (1.01-1.51)	.04	
Patient skills in self-management of their health (GPs perceived): low (0)-high (100), mean (SD)		48.8 (14.7)	49 (15.6)	1.00 (0.98-1.02)	.93	
**Had a connected health or wellness device, n (%)**						.10
	No	28 (62.2)	62 (48.1)	Ref		
	Yes	17 (37.8)	67 (51.9)	1.78 (0.90-3.62)		
**Had an mHealth app, n (%)**						.39
	No	13 (28.9)	29 (22.5)	Ref		
	Yes	32 (71.1)	100 (77.5)	1.40 (0.64-2.98)		
Facilitators: perceptions of GPs of the benefits of mHealth apps and devices for patients, caregivers, their own clinical practice, and GP-perceived drivers for mHealth apps and devices implementation in their medical practice^c^, mean (SD)		49.6 (18.1)	59.9 (15.2)	1.04 (1.02-1.06)	<.001	
Obstacles: perceptions of GPs of risks for the patient and barriers for the GPs practice^c^, mean (SD)		59 (15.8)	52.4 (15.3)	0.97 (0.95-0.99)	.02	
Perceptions of GPs of the usefulness of mHealth apps and devices certification^c^, mean (SD)		63 (15.4)	64.6 (15.3)	1.01 (0.98-1.03)	.54	
Perceptions of GPs of the importance of the involvement of health-related organizations and stakeholders in promoting the use of mHealth apps and devices in general medicine^c^, mean (SD)		61.5 (21.3)	65.7 (23.2)	1.01 (0.99-1.02)	.29	
Perceptions of GPs of the importance of the involvement of field-based actors in the construction of mHealth apps and devices^c^, mean (SD)		70.7 (19.8)	77.2 (19.6)	1.02 (1.00-1.03)	.06	
Perceptions of GPs of the utility of validating mHealth apps and devices using randomized studies (evidence-based medicine)^c^, mean (SD)		76.3 (24.7)	82.8 (19.9)	1.01 (1.00-1.03)	.08	

^a^OR: odds ratio.

^b^Ref: reference.

^c^Scores ranging from 0 to 100, with 100 representing the greatest perceived benefit or driver, risk or barrier, involvement, or utility, as applicable.

### Multivariate Binomial Logistic Regression

Table 4 shows the results of the multivariate binomial logistic regression after stepwise selection. Factors associated with the willingness of GPs to prescribe mHealth apps and devices were as follows: having attended a continued education program during the previous year (OR 6.17, 95% CI 1.52-28.72); their perception that overall their patient base has a good command of the French language (OR 1.45, 95% CI 1.13-1.88); their perception that mHealth apps and devices could bring benefits to the patient and their own medical practice; and their perception of drivers for mHealth apps and devices implementation in their medical practice (OR 1.04, 95% CI 1.01-1.07); and a strong perception of the importance of validating mHealth apps and devices through randomized studies (evidence-based medicine; OR 1.02, 95% CI 1.00-1.04). In contrast, older age (OR 0.95, 95% CI 0.91-0.98), being a female GP (OR 0.26, 95% CI 0.09-0.69), and the perception of greater risks for the patient and barriers to their own medical practice (OR 0.96, 95% CI 0.94-0.99) were associated with the unwillingness of GPs to prescribe mHealth apps and devices (Table 4). This model had a very good fit and prediction properties, with an area under the receiver operator characteristic curve of 0.825 (excellent classification performance) and a McFadden pseudo-*R*^2^ value of 0.28 (very good fit). Interactions between gender and facilitators and obstacles were not significant.

**Table 4:** Results of multivariate logistic regression (after the stepwise procedure) to explain the willingness of general practitioners (GPs) to prescribe mobile health (mHealth) apps and devices to their patients (N=174).

Variables		aOR^a^ (95% CI)	*P* value
Age of GPs (years)		0.95 (0.91-0.98)	.003
**Gender of GPs**			
	Male	Ref^b^	N/A^c^
	Female	0.26 (0.09-0.69)	.008
**Participation in a continued training program during the previous year**			
	No	Ref	N/A
	Yes	6.17 (1.52-28.72)	.01
**Participation in a peer group during the previous year**			
	No	Ref	N/A
	Yes	2.32 (0.94-6.01)	.07
**Subscription to a professional magazine**			
	No	Ref	N/A
	Yes	2.41 (0.85-6.89)	.10
Patient base command of the French language (GP perceived)		1.45 (1.13-1.88)	.004
Facilitators: perceptions of GPs of benefits of mHealth apps and devices for patients, caregivers, their own clinical practice, and GP-perceived drivers for mHealth apps and devices implementation in their medical practice		1.04 (1.01-1.07)	.003
Obstacles: perceptions of GPs of risks for the patient and barriers for the GP practice		0.96 (0.94-0.99)	.01
Perceptions of GPs of the importance of validating mHealth apps and devices using randomized studies (evidence-based medicine)		1.02 (1.00-1.04)	.047

^a^aOR: adjusted odds ratio.

^b^Ref: reference.

^c^N/A: not applicable.

## Discussion

### Principal Findings

The objective of this study is to understand the factors influencing the willingness of French GPs to prescribe mHealth apps and devices. Our results highlighted that the several factors involved were as follows: sociodemographic characteristics of GPs, especially age and gender; factors linked to continued education, patient base–related characteristics, especially perceptions of GPs of their patient base’s command of the French language; and factors linked to perceptions of GPs regarding mHealth apps and devices (benefits, drivers, risks, and barriers) and the perceived importance of clinical validation.

The health-related dimensions for which GPs were very willing to prescribe mHealth apps and devices were diabetes, asthma (ie, chronic diseases) and addictions, physical activity, and vaccination (ie, primary prevention) reflecting findings in the literature [16,18]. GPs were more likely to be willing to prescribe mHealth apps and devices in medical fields where they are already numerous and mHealth apps and devices that have already been clinically validated [13]. This result underlines the feasibility of the potential prescription of mHealth apps and devices. On the contrary, this finding may not be very revealing in terms of therapeutic areas where patients might need the most support.

This study reflects the previous findings about the importance of GP-perceived sociodemographic profiles of patients as a parameter in determining the integration of mHealth apps and devices into current clinical practice [22,39]. An Australian study showed that GPs who had been working longer were less willing to prescribe mHealth apps and devices [16], and a German study corroborated this fact as they showed that younger GPs saw mHealth apps more favorable [28], which reflects our findings here. One possible explanation for this finding is that younger GPs are more technologically savvy. In that sense, a French Barometer survey showed that physicians tended to prefer prescribing mHealth apps and devices to adolescent patients, professionally active patients, and to technologically savvy patients [39]. Furthermore, the fact that female GPs in this study were less willing to prescribe mHealth apps and devices than their male counterparts reflects the importance of the issue of gender in the appropriation of new technologies. Indeed, several studies on general populations have already shown that men are more inclined to use mHealth apps and devices than women [40,41], even if this association remains unclear, as other studies have shown that women are more inclined to use mHealth apps and devices [42-44]. However, we found no information that specifically concerned physicians regarding this issue. Concerning the ease of communication GPs have with their patients, in this study, we found that GPs who perceived their patient base to have a good command of French were more willing to prescribe them mHealth apps and devices. Several studies have highlighted the importance of a patient’s digital health literacy level and the difficulties faced in implementing mHealth apps and devices for both patients with low levels of eHealth literacy [16,17] and patients showing reluctance [13,17]. These findings highlight the possibility of a second-order digital divide, which is not a divide in terms of access to the internet and smartphones, rather a divide in the use of mHealth apps and devices, which in turn can widen the gap in health inequalities [45,46]. In contrast, patients’ good command of the French language could be a predictor of their ability to use mHealth apps and devices and therefore may influence the decision of GPs to prescribe these types of interventions.

Our study highlights the important role that individual perceptions of mHealth apps and devices play in the willingness of GPs to use them in clinical practice. More specifically, our multivariate model highlighted that perceptions of the benefits, drivers, risks, and barriers of mHealth apps and devices were linked to the willingness of GPs to prescribe them, confirming the results of the qualitative study, which served as the basis for the construction of the questionnaire [23]. Consistent with our study, a Malaysian study found that GP-perceived benefits of mHealth apps (performance expectancy) were associated with the willingness of GPs to recommend them to their patients [27]. The aforementioned Australian study indicated different ways to encourage GPs to adopt mHealth apps and devices [16]. In particular, the need for training in mHealth apps and devices, the possibility of obtaining a list of safe and effective mHealth apps and devices that have been validated by a health authority, and access to detailed descriptions of mHealth apps and devices. Thus, only 22% of German GPs felt able to advise mHealth apps to their patients [28]. These results reflect the findings of this study, as GPs reported having a low level of knowledge regarding mHealth devices and a high perception level regarding the usefulness of a specific software aid that could help them when prescribing mHealth apps and devices [16].

### The Need for an mHealth App and Device Prescription Software Aid

Public and private initiatives have led to the creation of comprehensive lists of a number of mHealth apps and devices currently available. In the United Kingdom, 2 initiatives have been implemented to create a library of health apps to help patients navigate the various options available to them. First, PatientView was developed in 2013 by user groups and incorporated a visible app user rating system [47]. Second, the National Health Service Apps Library is a nationwide initiative developed by the National Health Service [48]. In the United States, an independent, private platform was created in 2009 for health care professionals based on the experience and opinions of their peers [49]. This platform led to the development of a specific app, called iPrescribeApps, which aids physicians in prescribing suitable mHealth apps and devices for their patient-specific medical conditions [50]. In Catalonia, we can also notice the platform AppSalut that references mHealth apps that have obtained accreditation in terms of technology, usability, security, and reliability (regarding medical content) [5].

An Australian before-and-after intervention pilot study aimed to investigate the feasibility of prescribing mHealth apps in general practice. The 36 GPs included were given a prescription guide for 6 apps (description of the app, download instructions, and cost). Video presentations for the 6 apps can also be found using the download instructions. After 2 months, the video presentation of one of the apps, randomly selected, was sent to each physician in the study just to remind the GP. The median number of apps prescribed before and after the intervention was almost quadrupled. However, the video presentations were not associated with this increase, highlighting the importance of having a prescription guide to prescribe mHealth apps and devices [51].

Given our study’s findings and the initiatives and the literature on mHealth apps and devices described earlier, it would appear that there is great demand by GPs for an mHealth apps and devices prescription software aid. Such an aid would represent a real driver for the implementation of mHealth apps and devices in medical practice in France and would merit being developed, provided that clinical evaluation criteria of health-related organizations and stakeholders and the protection of personal data were considered.

### The Need for Training in mHealth

In this study, GPs reported not being sufficiently familiar with mHealth apps and devices. Training in mHealth would provide GPs with sufficient knowledge and confidence to prescribe mHealth apps and devices [52]. This issue was raised in a Dutch study [17], which included 621 GPs. Almost half of the participants declared their desire for remote learning (webinars, podcasts, etc) compared with only 12% who preferred face-to-face training [17]. It would be interesting to interview French GPs about this issue and ask their opinions about which training content would be most suitable to help them integrate mHealth into their medical practice. Governments, health systems, and authorities should provide digital health education to GPs [52] via continuing education programs and medical curricula. Training could be provided jointly by health authority mHealth referents, mHealth referent GPs, mHealth researchers, and developers of mHealth app and device national libraries.

### Issues Surrounding Certification, Data Privacy, and Development of mHealth Apps and Devices

As shown here and highlighted in several other studies, GPs are concerned about the protection of personal data and the reliability of mHealth apps and devices [13,17,21,22,28]. Indeed, the willingness and unwillingness of GPs to prescribe mHealth apps and devices reported the importance of certifying mHealth apps and devices by independent public bodies and the irrelevance, in their opinion, of certification by private health companies. This finding is corroborated by a study of different French physician organizations that found that three-quarters of those questioned reported that they trusted certification by a learned society or a health authority as opposed to only 2% who trusted certification by a private company [39]. In this study, GPs strongly expressed the need for field-based actors (patients, physicians, and academic researchers) to be involved in the development of mHealth apps and devices. However, this factor was not significantly associated with the willingness of GPs to prescribe mHealth apps and devices in the multivariate analysis.

### The Need for Clinical Validation of mHealth Apps and Devices

For reimbursement by health insurance to become a possibility, it is essential that clinical validation—ideally by randomized studies (evidence-based medicine)—be performed. In this study, clinical validation appeared to be an essential element in the willingness of GPs to prescribe mHealth apps and devices. A 2018 overview of systematic reviews of randomized clinical trials focusing on mHealth apps showed that only 22 apps, most focusing on diabetes, obesity, and mental health, had been clinically validated. However, most of these 22 apps were clinically validated in pilot studies with small sample sizes, thereby limiting the validity of the results [18]. Clinical validation of mHealth apps and devices is a real challenge and deserves to be integrated in a more systematic fashion in health research projects.

### Issues Surrounding Care, Compensation, and Financial Incentives in Terms of mHealth Apps and Devices

The notion of covering the cost of mHealth apps and devices through health insurance of patients was an important point for the GPs in this study, as was the possibility of health authorities providing financial incentives for GPs to prescribe mHealth apps and devices. However, neither element was directly associated with the willingness of GPs to prescribe mHealth apps and devices in the multivariate analysis. In a descriptive way, our results showed that having to provide support to patients in the use of mHealth apps and devices—thereby leading to a longer working time—appeared to be the major perceived obstacle perceived by GPs. This reflects the literature that mentions the desire of GPs for financial compensation for the time spent (during and outside consultation) both processing information coming from mHealth apps and devices and training themselves and their patients in the use of mHealth apps and devices [13,16,17,22]. Studies have also reported the problem of the costs of mHealth apps and devices [13,16] and the lack of reimbursement [13] for these costs as obstacles to the prescription of mHealth apps and devices.

### Limitations

We decided to oppose, from the perspective of behavior change, in this study 2 profiles of GPs—those willing to mHealth apps and devices prescription and those not as in France prescription of mHealth apps and devices, especially in general medicine, which is not integrated in current practice. Then the variable willingness to prescribe was dichotomized; thus, we lost the information regarding the amount of mHealth apps and devices that GPs were willing to prescribe.

At the epistemological level, this study adopts a comprehensive approach that focuses on understanding the psychosocial processes involved in the initiation or noninitiation of a behavior and the meaning that individuals give to it. This approach is important for understanding behavior toward a phenomenon (in our case, the willingness or unwillingness to prescribe mHealth apps and devices). We did not base our study on registers or sampling techniques that ensure the representativeness of the French population of GPs. However, with the comprehensive approach, this study provides interesting elements to better understand the obstacles and facilitators of GPs’ willingness to prescribe mHealth apps and devices to their patients. The study was not intended to be representative but sought to confirm the role of various factors associated with the willingness of GPs to prescribe mHealth apps and devices. Although the ratio of male to female GPs in this study reflects national numbers, GPs in this study were a little younger (mean age 45.1 (SD 13) vs 50.4 years at the national level) [53] and the patient base seemed to also be little younger compared with French national figures [54]. This may have resulted in a slight overestimation of GPs’ willingness to prescribe mHealth apps and devices. In addition, our sample size was relatively small, and we were unable to obtain the response rate given our methodology for administering the questionnaires, which may question the representativeness of the responding GPs. In this study, we grouped willingness (or unwillingness) to prescribe mHealth apps or connected health and wellness devices in the same indicator, which could be interesting in further studies to investigate if there are differences in factors associated with mHealth apps prescription and connected health and wellness devices prescription. The willingness (or unwillingness) to prescribe mHealth apps and devices grouped several health categories, and further studies should be conducted to investigate whether the identified factors differ between these different health conditions.

Given our sample size and principal component analysis, we created indicators that aggregated several perceptions GPs have toward mHealth apps and devices, but we cannot identify the individual factors that have a significant impact. In France, the prescription of mHealth apps and devices is not integrated in clinical routine; we then investigated obstacles and facilitators perceived rather than experienced. Further studies need to be conducted after the implementation of mHealth apps and devices in general medicine to investigate obstacles and facilitators experienced. In this study, we focused on the perceptions of GPs, as they are the essential link in the patient’s care pathway. Compared with GPs, it could also be interesting to investigate the perceptions of specialist physicians, as it can be assumed that they may have a different practice and a different relationship with their patients.

### Conclusions

To conclude, mHealth apps and devices represent an important dimension in general practice consultations that can complement other GP treatment methods. GPs in this study seemed inclined to fully integrate mHealth apps and devices into their practice, especially if they have access to tools to help them navigate their way in the field of digital health, similar to those that already exist for the prescription of drugs. Such tools should provide information on the benefits of mHealth apps and devices both for the GP practice and for the patient; the pros and cons of mHealth apps and devices; and data on how mHealth apps and devices are developed, validated, and certified.

Public authority–based initiatives for the certification of mHealth apps and devices are very important for mHealth apps and devices to become accepted in general medicine and must be extensively implemented. Clinical validation of mHealth apps and devices through scientific studies needs to be performed on a larger scale, not only with pilot studies. Indeed, validation should be integrated more systematically into health research projects. Training courses specifically designed to provide support GPs in fully integrating mHealth apps and devices into their practice are also indispensable. Such training, in turn, would ensure that GPs could provide the best support possible in terms of mHealth apps and devices use to their patients.

## Appendix

Multimedia Appendix 1 Checklist for Reporting Results of Internet E-Surveys.

Multimedia Appendix 2 The ApiAppS questionnaire.

Multimedia Appendix 3 Perceptions of general practitioners regarding mobile health.

Multimedia Appendix 4 Proportion of general practitioners (GPs) willing to prescribe mHealth apps and devices according to the 12 different health dimensions included in this study (N=129; GPs who declared their willingness to prescribe mHealth apps and devices).

## Abbreviations

GP: general practitioner
mHealth: mobile health
OR: odds ratio
