# Supplementary material for: Willingness of French General Practitioners to Prescribe mHealth Apps and Devices: Quantitative Study
Source: JMIR Mhealth Uhealth. 2022 Feb 11;10(2):e28372. doi: 10.2196/28372 (PMC9491832; doi:10.2196/28372)
Supplement: Multimedia Appendix 1 [file mhealth_v10i2e28372_app1.docx]

Checklist for Reporting Results of Internet E-Surveys (CHERRIES)

| ***Item Category*** | ***Checklist Item*** | ***In this study…*** |
| --- | --- | --- |
| **Design** | Describe survey design | GPs were recruited through several academic department of general practice of several medical faculties in France (Lyon, Nice, Rouen) and also from mailing lists of academic GPs, healthcare professional associations, and social and professional networks. |
| **IRB (Institutional Review Board) approval and informed consent process** | IRB approval | Ethical approval was obtained from the French Institute of Medical and Health Research Ethics Committee (IORG0003254, FWA00005831) and Institutional Review Board (IRB00003888) (opinion number 18-499). |
|  | Informed consent | Informed consent was provided at the beginning of the questionnaire (references to French legislation on the processing of personal data). The time needed to answer this questionnaire was announced to people as 15 minutes (22 items). Data collected via the questionnaire were confidential and only analyzed for research purposes. |
|  | Data protection | No personal information was collected, all research data were collected, stored and analyzed anonymously for research purposes only. |
| **Development and pre-testing** | Development and testing | We constructed an online self-administered questionnaire on the basis of the results of the qualitative study concerning attitudes of GPs toward prescription of mHealth apps and devices [23] and elements from the literature concerning mHealth apps and devices in current clinical practice. The questionnaire was pre-tested with eight GPs regarding the understanding of the different items in the questionnaire, the researchers of the study tested the technical functionalities of the questionnaire. These two test phases made it possible to correct, where necessary, the layout of the questionnaire (spelling, fonts, order of questions, etc.) as well as the obligatory or non-obligatory nature of each question. The questionnaire was then distributed from June 2019 to December 2019 to GPs recruited through several academic department of general practice of several medical faculties in France (Lyon, Nice, Rouen) and also from mailing lists of academic GPs, healthcare professional associations, and social and professional networks. |
| **Recruitment process and description of the sample having access to the questionnaire** | Open survey versus closed survey | Participation to the study required thus a survey link, this link headed toward the questionnaire (on LimeSurvey platform). Information was provided about the time needed to fill in the questionnaire (about 15 minutes, 22 items), reminders about the rights of research participants under French law (anonymity, confidentiality, processing of data for research purposes, right of access and rectification of data), and contact e-mail addresses for the researchers in charge of the study were given. |
|  | Contact mode | The questionnaire was distributed to GPs recruited through several academic department of general practice of several medical faculties in France (Lyon, Nice, Rouen) and also from mailing lists of academic GPs, healthcare professional associations, and social and professional networks. |
|  | Advertising the survey | Survey was announced through mailing lists: several French academic department of general practice (Lyon, Nice, Rouen), academic GPs. Survey was also announced in health care professionals associations, social and professional networks. |
| **Survey administration** | Web/E-mail | The questionnaire was accessible via a link to an online questionnaire platform (LimeSurvey).  The input of the answers was automatic in the LimeSurvey platform, an Excel database with the participants' answers was generated. |
|  | Context | The questionnaire was accessible via a link to an online questionnaire platform (LimeSurvey). |
|  | Mandatory/voluntary | It was a voluntary survey. |
|  | Incentives | No incentives offered. |
|  | Time/Date | June 2019 to December 2019 |
|  | Randomization of items or questionnaires | No |
|  | Adaptive questioning | Yes, there are filter questions in the questionnaire (e.g. having answered "yes" to question 14. Do you own one or more connected health object(s)?] to be offered the following question: Do you use this/these object(s)? for strictly personal use; for strictly professional use; for both personal and professional use. And in the same way for questions 16 and 17 on health apps. |
|  | Number of Items | 22 items |
|  | Number of screens (pages) | 19 pages |
|  | Completeness check | All questionnaire items were required to be completed. |
|  | Review step | Once validated, the answers to the questions could not be changed. |
| **Response rates** | Unique site visitor | Not available. |
|  | View rate (Ratio of unique survey visitors/unique site visitors) | Not available |
|  | Participation rate (Ratio of unique visitors who agreed to participate/unique first survey page visitors) | Not available |
|  | Completion rate (Ratio of users who finished the survey/users who agreed to participate) | Only fully completed questionnaires were analyzed, 174 GPs among 226 GPs who answer the first question of the survey (regarding their status) fully completed the questionnaire (174/226, 77%). |
| **Preventing multiple entries from the same individual** | Cookies used | No cookies were used. |
|  | IP check | No method was used to identify potential duplicates. |
|  | Log file analysis | No other techniques used. |
|  | Registration | Not applicable. |
| **Analysis** | Handling of incomplete questionnaires | We analyzed only completed questionnaires, excluding questionnaires that had missing data due to the responder stopping early and leaving the website. |
|  | Questionnaires submitted with an atypical timestamp | Data not available. |
|  | Statistical correction | No statistical correction was made. |
