# Supplementary material for: Willingness of French General Practitioners to Prescribe mHealth Apps and Devices: Quantitative Study
Source: JMIR Mhealth Uhealth. 2022 Feb 11;10(2):e28372. doi: 10.2196/28372 (PMC9491832; doi:10.2196/28372)
Supplement: Multimedia Appendix 2 [file mhealth_v10i2e28372_app2.docx]

**The ApiApps questionnaire**

1. Are you…
   - An intern in general medicine
   - A general practitioner practising only as an employee
   - A general practitioner in private practice only
   - A general practitioner practising both as an employee and in private practice
   - An intern or doctor practising a specialty other than general medicine
2. Please indicate your age
3. You are
   - A man
   - A woman
   - Other
4. In the past year, have you attended any training related to your practice?
   - Yes
   - No
5. In the past year have you participated in a peer group?
   - Yes
   - No
6. Do you subscribe to one or more professional journals?
   - Yes
   - No
7. Do you belong to one or more social networks for doctors?
   - Yes
   - No
8. In the last month and in the context of your main activity, in which age group was the largest proportion of your patient base?
   - 0 to 12 years old
   - 13 to 24 years old
   - 25 to 44 years old
   - 45 to 69 years old
   - 70 years old and over
   - My current practice is too varied or recent for me to answer
9. For each of the following scales, please indicate what you think best describes your patient base, overall and on average, over the last month and in the context of your main activity (from 0 to 6)

|  | 0 | 1 | 2 | 3 | 4 | 5 | 6 |  |
| --- | --- | --- | --- | --- | --- | --- | --- | --- |
| Socially deprived |  |  |  |  |  |  |  | Socially favoured |
| rural |  |  |  |  |  |  |  | urban |
| not at all at ease with French |  |  |  |  |  |  |  | completely at ease with French |

1. The following statements relate to your perception of your patient base over the past month and in the context of your main activity.

|  | Strongly disagree | 2 | 3 | 4 | 5 | 6 | 7.  Totally agree |
| --- | --- | --- | --- | --- | --- | --- | --- |
| For the most part, my patients are attentive to the prevention of risks to their health |  |  |  |  |  |  |  |
| For the most part, my patients with chronic conditions manage their disease well |  |  |  |  |  |  |  |
| For the most part, my patients manage their health independently |  |  |  |  |  |  |  |
| For the most part, my patients are able to assess whether health-related information is reliable or not |  |  |  |  |  |  |  |

1. During a consultation, on average, how much time do you spend on promoting health behaviours?

| 0% | 10% | 20% | 30% | 40% | 50% | 60% | 70% | 80% | 90% | 100% |
| --- | --- | --- | --- | --- | --- | --- | --- | --- | --- | --- |
|  |  |  |  |  |  |  |  |  |  |  |

**Connected devices and health applications**

**IMPORTANT: We exclude from the OCA any object or application designed for the practice of telemedicine**

A connected object is a wireless object, whose information can be read on the object itself and/or sent to a computer, tablet, iPad, or smartphone. For example, a connected pedometer tracks the number of steps you take like a traditional pedometer and allows you, if you wish, to have a summary of your activity in the form of a graph on your smartphone or computer. It can also be a connected scale, an activity tracker watch, a blood pressure monitor or a connected blood glucose meter.

1. Do you understand?
   - Yes
   - No

An application is a program that can be downloaded to a mobile device such as a smartphone or tablet. These applications then analyse this information to give you personalised advice. For example, some of the above applications are installed on your mobile device (smartphone, tablet...) and allow you to record data such as your weight, the composition of your meals and the number of steps you take. They can also be applications such as menstrual calendar, vaccine tracking, etc.

1. Do you understand?
   - Yes
   - No
2. Do you own one or more connected health objects?
   - Yes
   - No
3. Do you use this/these object(s)?
   - For strictly personal use
   - For strictly professional use
   - For both personal and professional use
4. Do you have one or more health applications?
   - Yes
   - No
5. Do you use this/these application(s)?
   - For strictly personal use
   - For strictly professional use
   - For both personal and professional use

**From now on we will use the acronym "OCA" to refer to Connected Objects and Health Applications.**

1. The following statements relate to your perception of the use of connected objects and health applications by users of the health system.

|  | 1 strongly disagree | 2 | 3 | 4 | 5 | 6 | 7  totally agree |
| --- | --- | --- | --- | --- | --- | --- | --- |
| OCAs strengthen people's involvement in managing their health |  |  |  |  |  |  |  |
| OCAs are simply an extension of tools that already existed before |  |  |  |  |  |  |  |
| OCAs strengthen people's involvement in managing their health |  |  |  |  |  |  |  |
| I am concerned about the commercial use that may be made of the personal data of OCA users |  |  |  |  |  |  |  |
| Using OCAs leads to a dehumanisation of the doctor-patient relationship |  |  |  |  |  |  |  |
| OCAs can lead to a tendency to self-medicate that is problematic for patients |  |  |  |  |  |  |  |
| The use of OCAs causes additional anxiety in patients due to the amount of information available |  |  |  |  |  |  |  |
| OCAs promote access to care for geographically isolated people |  |  |  |  |  |  |  |
| OCAs promote access to care for socially isolated people |  |  |  |  |  |  |  |

1. The following statements relate to the use of OCAs for follow-up in general practice.

|  | 1  strongly disagree | 2 | 3 | 4 | 5 | 6 | 7  totally agree |
| --- | --- | --- | --- | --- | --- | --- | --- |
| I think I know enough about OCAs to decide whether to recommend them to my patients |  |  |  |  |  |  |  |
| I need to test an OCA before I recommend it to my patients |  |  |  |  |  |  |  |
| Recommending OCAs is part of my role as a GP |  |  |  |  |  |  |  |
| More of my patients would use OCAs if I recommended them |  |  |  |  |  |  |  |
| I feel ready to spend time adapting my practice to the increasing use of OCAs |  |  |  |  |  |  |  |
| Thanks to the OCAs I will have objective information about the health of my patients |  |  |  |  |  |  |  |
| OCAs can help me to link with other professionals involved in the care of my patients (e.g. physiotherapist) |  |  |  |  |  |  |  |
| OCAs for medical practice can be used by health authorities to monitor the activity of doctors |  |  |  |  |  |  |  |
| OCAs are sometimes an interesting alternative to prescribing a drug |  |  |  |  |  |  |  |
| OCAs improve doctors' relationships with their patients |  |  |  |  |  |  |  |
| The use of OCAs as part of medical follow-up should be accompanied by the doctor |  |  |  |  |  |  |  |
| OCAs generate a lot of information that cannot be processed by doctors |  |  |  |  |  |  |  |
| A software that would recommend OCAs adapted to my patients' pathologies would be useful |  |  |  |  |  |  |  |

1. The following statements relate to your views on the use of connected objects and health applications in general practice, particularly in relation to support for carers. OCAs are relevant to...

|  | 1  strongly disagree | 2 | 3 | 4 | 5 | 6 | 7  totally agree |
| --- | --- | --- | --- | --- | --- | --- | --- |
| Facilitating communication with carers |  |  |  |  |  |  |  |
| Improving the quality of life of carers |  |  |  |  |  |  |  |
| Improving the health management of non-autonomous children and adults by their carers |  |  |  |  |  |  |  |

1. We are going to ask you to imagine whether you would be likely (if it were possible today) to:

- put up posters or leaflets in your waiting room promoting an OCA,

- put up posters or leaflets in your waiting room promoting an OCA, - orally recommend an OCA during a consultation,

- prescribe the use of an OCA on a prescription.

Please indicate for each topic whether you would be willing to display, recommend or prescribe an OCA. This is a multiple choice questionnaire, you can select all the options that apply.

If you would not be willing to display, recommend or prescribe an OCA, please tick the box "none of the above".

|  | None of these options | Display | Recommend | Prescribe |
| --- | --- | --- | --- | --- |
| First aid |  |  |  |  |
| Sexual and reproductive health |  |  |  |  |
| Asthma and allergies |  |  |  |  |
| Well-being and mental health |  |  |  |  |
| Vaccination |  |  |  |  |
| Diabetes |  |  |  |  |
| Addictions |  |  |  |  |
| Dermatology |  |  |  |  |
| Physical activity |  |  |  |  |
| Nutrition |  |  |  |  |
| Dental health |  |  |  |  |
| Support for caregivers |  |  |  |  |

1. If you were to prescribe OCAs as part of your medical practice in the future, how useful do you think the following suggestions would be?

|  | 1.Would be completely unnecessary | 2 | 3 | 4 | 5 | 6 | 7. Would be very necessary |
| --- | --- | --- | --- | --- | --- | --- | --- |
| Have a detailed prescription guide |  |  |  |  |  |  |  |
| Certification by a college of doctors |  |  |  |  |  |  |  |
| Certification by independent experts |  |  |  |  |  |  |  |
| A label from a patient association |  |  |  |  |  |  |  |
| A university label |  |  |  |  |  |  |  |
| Certification by an ethical committee |  |  |  |  |  |  |  |
| Certification by private health companies |  |  |  |  |  |  |  |
| The participation of doctors in the construction of the application and the development of its content |  |  |  |  |  |  |  |
| The participation of patients in the construction of the application and in the development of its content |  |  |  |  |  |  |  |
| The participation of academic researchers in the construction of the application and the development of its content |  |  |  |  |  |  |  |
| Clinical validation through randomised trials (EBM) |  |  |  |  |  |  |  |
| Coverage by the French Social Security |  |  |  |  |  |  |  |
| Coverage by complementary health insurance firms |  |  |  |  |  |  |  |
| A financial incentive from the French Health Insurance (remuneration based on public health objectives) |  |  |  |  |  |  |  |
| A recommendation by a health authority |  |  |  |  |  |  |  |
