# Supplementary material for: Willingness of French General Practitioners to Prescribe mHealth Apps and Devices: Quantitative Study
Source: JMIR Mhealth Uhealth. 2022 Feb 11;10(2):e28372. doi: 10.2196/28372 (PMC9491832; doi:10.2196/28372)

Figure S1. GPs’ perceptions regarding facilitators of mHealth apps and devices implementation in general medicine (both the potential perceived benefits of mHealth apps and devices and the GP-perceived levers to their implementation).


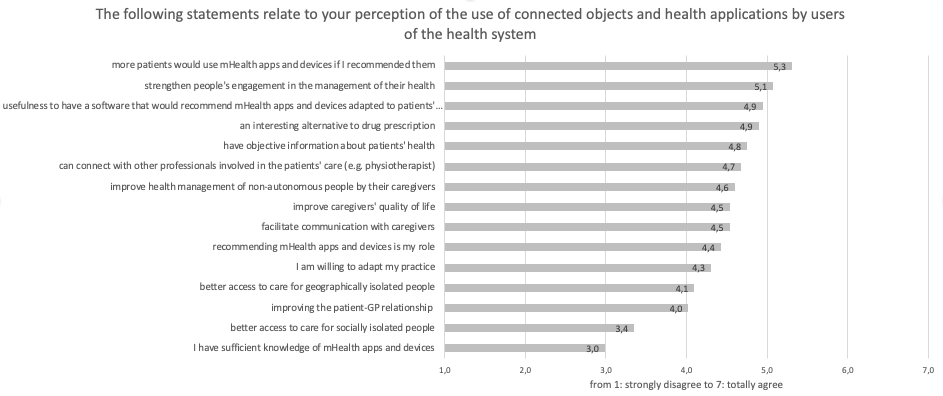


Figure S2. GPs’ perceptions regarding obstacles to mHealth apps and devices implementation in general practice (risks and barriers associated with the use of mHealth apps and devices).


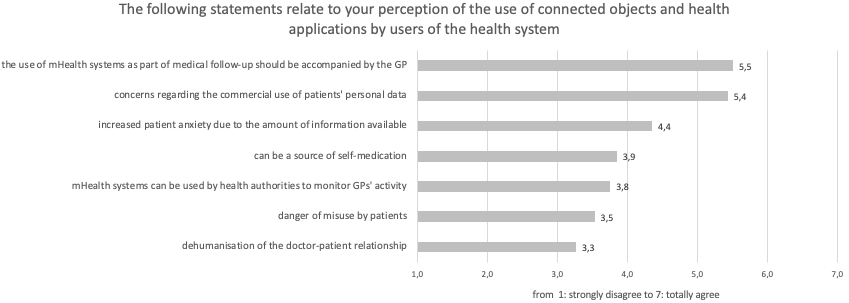


Figure S3. GPs’ perceptions regarding the importance of the implication of the following actors in the construction of mHealth apps and devices.


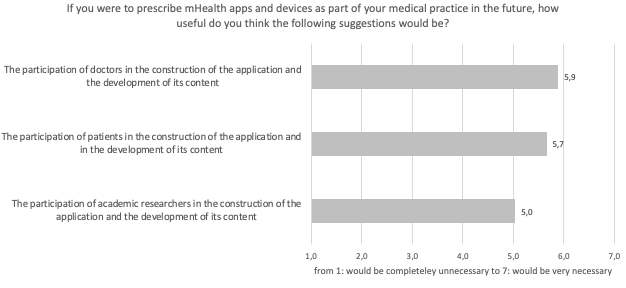


Figure S4. GPs’ perceptions regarding the importance of mHealth apps and devices certification.


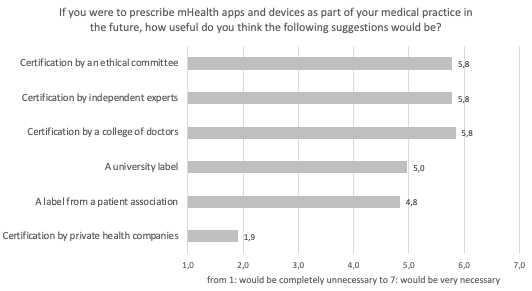


Figure S5. GPs’ perceptions regarding the importance of the implication of health-related organisations and stakeholders in promoting the use of mHealth apps and devices


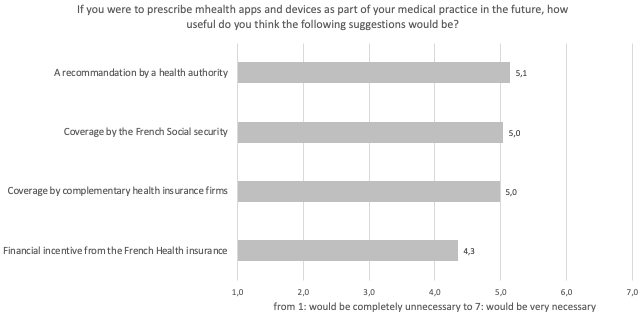

Supplement: Multimedia Appendix 3 [file mhealth_v10i2e28372_app3.docx]
