# Supplementary material for: Willingness of French General Practitioners to Prescribe mHealth Apps and Devices: Quantitative Study
Source: JMIR Mhealth Uhealth. 2022 Feb 11;10(2):e28372. doi: 10.2196/28372 (PMC9491832; doi:10.2196/28372)

Proportion of GPs willing to prescribe mHealth apps and devices according to the 12 different health dimensions included in this study (N=129, GPs who declared their willingness to prescribe mHealth apps and devices)


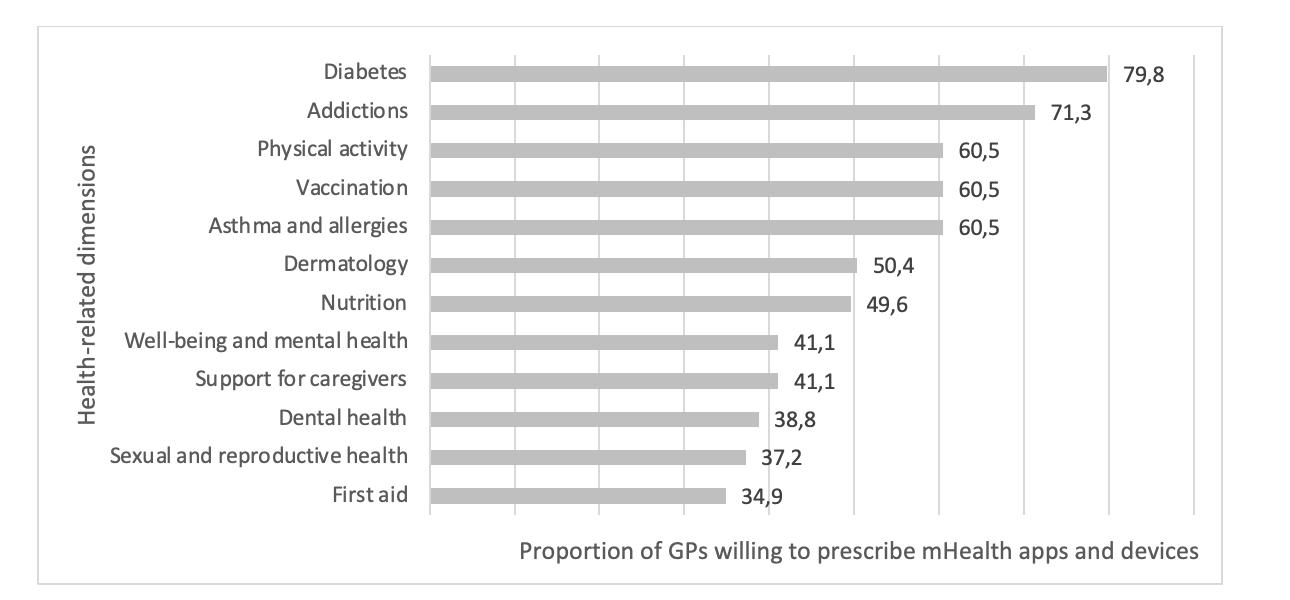

Supplement: Multimedia Appendix 4 [file mhealth_v10i2e28372_app4.docx]
